# ERCC1–XPF targeting to psoralen–DNA crosslinks depends on XPA and FANCD2

**DOI:** 10.1007/s00018-019-03264-5

**Published:** 2019-08-07

**Authors:** Mariangela Sabatella, Alex Pines, Jana Slyskova, Wim Vermeulen, Hannes Lans

**Affiliations:** 1 Department of Molecular Genetics, Erasmus MC, 3015 GE Rotterdam, The Netherlands; 2 Oncode Institute, Erasmus MC, 3015 GE Rotterdam, The Netherlands; 3 Present Address: CeMM Research Centre for Molecular Medicine of the Austrian Academy of Sciences, 1090 Vienna, Austria

**Keywords:** DNA damage response, Fanconi anemia, DNA repair, Xeroderma pigmentosum–Cockayne syndrome complex

## Abstract

The effectiveness of many DNA-damaging chemotherapeutic drugs depends on their ability to form monoadducts, intrastrand crosslinks and/or interstrand crosslinks (ICLs) that interfere with transcription and replication. The ERCC1–XPF endonuclease plays a critical role in removal of these lesions by incising DNA either as part of nucleotide excision repair (NER) or interstrand crosslink repair (ICLR). Engagement of ERCC1–XPF in NER is well characterized and is facilitated by binding to the XPA protein. However, ERCC1–XPF recruitment to ICLs is less well understood. Moreover, specific mutations in XPF have been found to disrupt its function in ICLR but not in NER, but whether this involves differences in lesion targeting is unknown. Here, we imaged GFP-tagged ERCC1, XPF and ICLR-defective XPF mutants to investigate how in human cells ERCC1–XPF is localized to different types of psoralen-induced DNA lesions, repaired by either NER or ICLR. Our results confirm its dependence on XPA in NER and furthermore show that its engagement in ICLR is dependent on FANCD2. Interestingly, we find that two ICLR-defective XPF mutants (R689S and S786F) are less well recruited to ICLs. These studies highlight the differential mechanisms that regulate ERCC1–XPF activity in DNA repair.

**Electronic supplementary material:**

The online version of this article (10.1007/s00018-019-03264-5) contains supplementary material, which is available to authorized users.

## Introduction

The heterodimeric ERCC1–XPF complex (also called ERCC1–ERCC4) is a structure-specific endonuclease that plays an essential role in multiple DNA repair pathways by incising DNA repair intermediate fork structures at the junction between single-stranded and double-stranded DNA. ERCC1–XPF excises bulky DNA lesions in nucleotide excision repair (NER) [1], unhooks interstrand crosslinks (ICLs), i.e., covalent linkages between two bases on opposite DNA strands, in interstrand crosslink repair (ICLR) [2–4], removes 3′ flaps of DNA intermediate structures in specific double-strand break (DBS) repair pathways [5] and cleaves 3′ overhangs from uncapped telomeres to shorten them [6]. Hereditary mutations in ERCC1–XPF cause several distinct human syndromes characterized by either (skin) cancer proneness, i.e., xeroderma pigmentosum (XP), developmental abnormalities and accelerated aging, i.e., XP combined with Cockayne syndrome (XPCS complex), or bone marrow failure and chromosome fragility, i.e., Fanconi anemia (FA) [1, 7–10]. The severe and pleiotropic symptoms associated with these syndromes highlight the fundamental role of the complex in promoting health by its essential role in different DNA repair processes.

The ERCC1–XPF complex was initially identified as an essential endonuclease in NER [1]. This DNA repair pathway removes many different types of DNA lesions that disrupt base-pairing, including photolesions induced by UV light and monoadducts and intrastrand crosslinks induced by widely used chemotherapeutic drugs such as mitomycin C (MMC), cisplatin and psoralens [11, 12]. NER is a multistep process consisting of the coordinated, sequential assembly of multiple repair enzymes guided by direct protein–protein and protein–DNA interactions. NER is initiated by two different damage sensing mechanisms. In global genome NER (GG-NER), lesions located anywhere in the genome are recognized by the UV-DDB and XPC–RAD23B–CETN2 complexes. In transcription-coupled NER, lesions located in the transcribed strand are sensed by stalling of elongating RNA polymerase II, which leads to the recruitment of the CSA, CSB and UVSSA proteins. Lesion recognition in both subpathways is followed by recruitment of the transcription factor TFIIH, which unwinds the DNA and verifies the presence of damage. TFIIH function is stimulated by the DNA damage-binding protein XPA [13], which together with the single-stranded DNA-binding protein RPA coordinates the positioning of the endonucleases ERCC1–XPF and XPG. XPG is first recruited through an interaction with TFIIH [14]. Next, ERCC1–XPF is recruited through a direct interaction between XPA and an XPA-binding region located within the central domain of ERCC1 [15–19]. XPF is the actual nuclease of this complex and incises the DNA 5′ to the lesion [20], after which XPG incises the DNA 3′ of the lesion. Following excision, the resulting 22–30 nt gap is filled by DNA synthesis and ligation.

In addition to NER, ERCC1–XPF is essential for the removal of ICLs. These are rare DNA modifications induced by either endogenous aldehydes [21] or by chemotherapeutic drugs such as MMC, cisplatin and psoralens [22]. ICLs are highly cytotoxic as they obstruct DNA strand separation, required for both replication and transcription. However, despite many studies focused on understanding how ICLs are removed from the genome, the molecular mechanism(s) of ICLR is not yet entirely clear. ICLR is a multistep, sequential assembly of repair enzymes and involves distinct subpathways depending on the cell cycle phase and type of ICL. Most ICLs are repaired during S phase, which requires the concerted action of different DNA repair systems, including the Fanconi anemia (FA) and homologous recombination (HR) repair pathways, NER and translesion synthesis (TLS). This replication-dependent ICLR is proposed to be initiated when two replication forks converge on an ICL. This leads to the helicase heterodimer FANCM/FAAP24-dependent recruitment of the FA-core complex and monoubiquitylation of the FANCD2–FANCI heterodimer [23, 24]. Experiments using *Xenopus laevis* egg extract and in vitro-modeled ICL-containing replication structures suggest that ERCC1–XPF is then recruited, depending on its interaction with the scaffold protein SLX4 that stimulates its function [2, 25, 26]. ERCC1–XPF incises the lagging strand to unhook the ICL, possibly together with another endonuclease or together with the exonuclease SNM1A that digests past the ICL [27]. The resulting single-stranded gap is filled by TLS and is used as homology template for repair of the remaining double-strand break by HR [28, 29]. The unhooked crosslink is likely repaired by NER. Certain types of ICLs, such as, for instance, induced by psoralens, appear to be preferentially unhooked by DNA glycosylase NEIL3-mediated cleavage of one of the two *N*-glycosyl bonds forming the crosslink, which circumvents the need for FA factors and incision of the DNA backbone [30]. Other forms of ICLR probably exist that are independent of replication, such as in slowly or nonreplicating cells, but these are only poorly understood. Replication-independent ICLR is proposed to involve GG-NER and/or TC-NER factors, likely depending on the type of crosslinked chemical, including ERCC1–XPF to unhook the ICL [31–35]. TLS then fills the resulting gap while a second round of NER removes the remaining ICL from the opposite DNA strand [22, 28].

Understanding how ERCC1–XPF is recruited to sites of DNA damage is necessary to understand how ERCC1–XPF protects against different types of DNA damage, including those induced by commonly used chemotherapeutics, how it helps to prevent cancer and how its inherited deficiency can cause different diseases. Previous imaging studies by us and others have highlighted the importance of XPA in regulating ERCC1–XPF recruitment to UV damage and have shown that patient-derived mutations within XPF can differentially affect its subcellular localization or recruitment to UV-induced DNA damage, explaining phenotypic differences observed in diseases associated with NER dysfunction, such as XP and XPCS complex [16, 17, 36, 37]. However, XPA is not implicated in replication-dependent ICLR and not much is known about how ERCC1–XPF is recruited to ICLs in human cells. Here we used imaging of wild-type and ICLR-defective XPF to investigate how ERCC1–XPF is distinctly recruited to psoralen–DNA crosslinks repaired by NER or by ICLR. Our results substantiate that FANCD2 is required for recruitment of the complex to ICLs and suggest that XPF ICLR-defective mutants are unable to efficiently associate with ICLs.

## Methods

### Cell culture and siRNA

U2OS cells expressing ERCC1–GFP, XPF–GFP and XPF-C236R, XPF-R689S and XPF-S786F were previously described [37, 38]. To knockout XPA, U2OS cells expressing ERCC1–GFP or XPF–GFP were transfected with pLentiCRISPR-V2 plasmid [39] encoding the sgRNA GGCGGCTTTAGAGCAACCCG, targeting the first exon of the *XPA* gene. Transfected cells were selected with puromycin and individual XPA KO clones were screened by immunoblot and sequencing and analyzed using Tracking Indels by DEcomposition as described [40]. All cell lines were cultured in standard condition: DMEM/F10 supplemented with 10% fetal calf serum (FCS) and 1% penicillin–streptomycin (PS) at 37 °C and 5% CO_2_. For siRNA treatment, cells were transfected using RNAiMax (Invitrogen) with control siRNA (Dharmacon, D-001210-05), siRNA targeting XPA (Dharmacon, MJAWM-000011) or FANCD2 (Dharmacon, D-016376-02), 48 h before UVC or 8-MOP treatment.

### Immunoprecipitation

Immunoprecipitation of chromatin-enriched nuclear extracts for mass spectrometry was performed as described [41]. ERCC1–GFP cells were seeded in 14-cm culture dishes and irradiated with 20 J/m^2^ UVC (254 nm lamp, Philips) or left untreated. After 1 h, cells were harvested by scraping in 3 ml cold PBS containing protease inhibitor cocktail (Roche), centrifuged for 10 min at 1500 rpm and washed again with PBS. Cell pellet was then incubated in HEPES buffer (10 mM HEPES, pH 7.6, 1.5 mM MgCl_2_, 10 mM KCl, 0.5 mM DTT, protease inhibitor cocktail) for 10 min on ice. Dounce homogenizer with a type A pestle was used to isolate the nuclei prior to centrifugation at 3000 rpm for 10 min at 4 °C. Next, cell pellets were resuspended in HEPES buffer (100 mM HEPES, pH 7.6, 1.5 mM MgCl_2_, 150 mM NaCl, 0.5 mM DTT, 25% glycerol, protease inhibitor cocktail) and subsequently dounced using a type B pestle. 25 U micrococcal nuclease (MNase, Sigma) was used to digest chromatin extracts for 1 h at 4 °C to obtain mononucleosomal size material by centrifugation at 15,000 rpm for 15 min.

Immunoprecipitation of benzonase-treated lysates was performed as described [42]. Briefly, cells were lysed using IP buffer (30 mM Tris, pH 7.5, 150 mM NaCl, 2 mM MgCl2, 0.5% Triton X-100, protease inhibitor cocktail (Roche)) supplemented with 250 U/mL Benzonase^®^ nuclease.

In both cases, GFP-Trap^®^_A beads (Chromotek) were used to immunoprecipitate GFP-tagged proteins. Cell lysate and immunoprecipitated samples were analyzed by immunoblot or mass spectrometry. For mass spectrometry, beads from UVC- and mock-treated cells were combined in a 1:1 ratio and label swapping was used to validate the biological findings and exclude contaminants.

### Immunoblot

Protein lysates were prepared by scraping cells in 2 × sample buffer (125 mM Tris–HCl, pH 6.8, 20% glycerol, 10% 2-β-mercaptoethanol, 4% SDS, 0.01% bromophenol blue) and boiled at 98 °C for 5 min. Proteins were separated by SDS-PAGE and transferred to a PVDF membrane (0.45 µm, Merck Millipore). Subsequently, membranes were blocked in 2% BSA and incubated with primary antibodies and secondary antibodies conjugated with CF IRDye 680 and 770 (Sigma) for 1 h or overnight. Primary antibodies used were anti-XPA (sc-853, Santa Cruz Biotechnology), anti-SNF2H (ab3749, Abcam), anti-Tubulin (T6074, Sigma), anti-FANCD2 (nb100-316, Novus Biologicals), anti-GFP (ab290, Abcam), anti-SLX4 (NBP1-28680, Novus Biologicals), anti-ERCC1 (ab129267, Abcam), anti-H2B (07-371, Millipore), and anti-RPA70 (2267, Cell signaling). Secondary antibodies were visualized using the Odyssey CLx Infrared Imaging System (LI-COR Biosciences).

### Clonogenic survival assays

To analyze the UV sensitivity of the ERCC1–GFP and XPF–GFP XPA-deficient cells, we seeded 500 cells in six-well plates in triplicate. After 24 h, cells were irradiated with the indicated doses of UV (254 nm UVC lamp, Philips). 5–7 days after irradiation, cells were fixed and stained with 50% methanol, 7% acetic acid, and 0.1% Brilliant Blue R (Sigma). Colonies were counted using the integrated colony counter GelCount (Oxford Optronix). The number of colonies in the non-treated cells was set to 100% and the number of colonies after UV treatment was normalized to the number of colonies in untreated samples and plotted as percentage survival.

### UV, psoralen treatment and immunofluorescence

To analyze the localization of proteins to LUD, cells seeded on coverslips were irradiated with 60 J/m^2^ (254 nm UVC lamp, Philips) through an 8-µm microporous filter (Millipore) and fixed with 2% paraformaldehyde supplemented with 0.1% Triton X-100, 1 h after irradiation. Next, cells were permeabilized with 0.1% Triton X-100 for 20 min, incubated with 0.07 M NaOH for 5 min (to visualize CPDs) and washed in PBS containing 0.15% glycine and 0.5% BSA before proceeding with immunofluorescence. Analysis of protein localization to psoralen-induced DNA damage was performed as described [43, 44]. Briefly, cells were seeded on coverslips and incubated with 50 µM 8-methoxypsoralen (8-MOP, Sigma) for 2 h. 8-MOP was locally activated in stripes along the cell nuclei by a 355-nm UVA laser coupled to a PALM laser dissection microscope (Zeiss) through a 40 × 0.60 NA Korr LD Plan Neofluar objective. Cells were then fixed with 2% paraformaldehyde supplemented with 0.1% Triton X-100 and washed in PBS containing 0.15% glycine and 0.5% BSA before proceeding with immunofluorescence. For immunofluorescence, cells were stained with primary antibodies for 2 h and then incubated with secondary antibodies conjugated to ALEXA fluorochromes 488, 555 and 633 (Invitrogen) for 1 h. Primary antibodies used were against: GFP (ab290, Abcam), CPD (CAC-NM-DND-001; Cosmo Bio), XPA (sc-853, Santa Cruz Biotechnology), FANCD2 (nb100-316, Novus Biologicals), SLX4 (NBP1-28680, Novus Biologicals), γH2AX (ab11174, Abcam or 05-636, Millipore), XPF (sc-136153, Santa Cruz Biotechnology). DAPI Vectashield (Vector Laboratories) was used to mount the coverslips. Cells were imaged using an LSM700 microscope equipped with a 40 × Plan-apochromat 1.3 NA oil immersion lens (Carl Zeiss). The software Image J was used to quantify fluorescence intensity. Fold accumulation was calculated as the ratio between fluorescence intensity at the site of damage and fluorescence intensity in the cell nucleus, measured in at least 60 cells per condition in two or more separate experiments. Statistical analysis of the differences in GFP intensity at sites of local damage was performed using an unpaired two-tailed Student’s *t* test.

### SILAC-based mass spectrometry analysis

For SILAC, cells were cultured in DMEM containing 10% dialyzed FBS (Gibco), 10% GlutaMAX (Life Technologies), penicillin/streptomycin (Life Technologies), unlabeled l-arginine–HCl and l-lysine–HCl (“light”) or ^13^C_6_,^15^N_4_l–arginine–HCl and ^13^C_6_,^15^N_2_l–lysine–2HCl (“heavy”) (Cambridge Isotope Laboratories). For the “Forward” experiment, immunoprecipitation extracts derived from “light”-labeled mock-treated cells were mixed with extracts derived from “heavy” UVC-treated (20 J/m^2^) cells; for the “Reverse” experiment, extracts derived from “light”-labeled UVC-treated (20 J/m^2^) cells were mixed with extracts derived from “heavy” mock-treated cells. After protein separation, SDS-PAGE gel was cut into 2-mm slices and subjected to in-gel reduction with dithiothreitol, alkylation with iodoacetamide (98%; D4, Cambridge Isotope Laboratories) and digested with trypsin (sequencing grade; Promega). Samples were analyzed by mass spectrometry using an Orbitrap Fusion™ Tribrid™ mass spectrometer and EASY-nLC™ 1000 (Thermo). MaxQuant software was used to analyze the data.

## Results

### ERCC1–XPF recruitment to sites of UV damage is XPA dependent

**Fig. 1:**
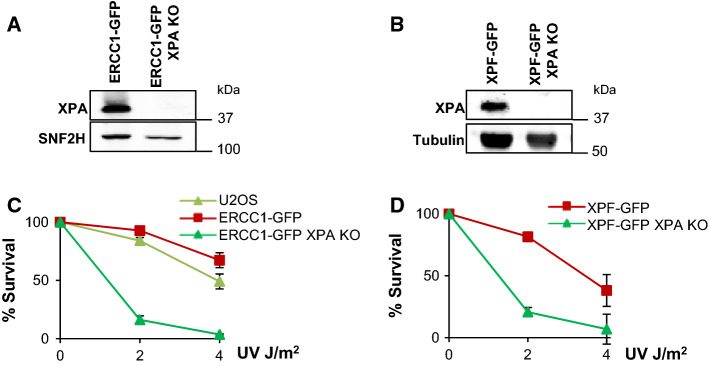
Generation of XPA knockout in ERCC1–GFP- and XPF–GFP-expressing cells. **a** Immunoblot showing XPA expression levels in XPA-proficient and XPA-knockout (XPA KO) U2OS cells expressing GFP-tagged ERCC1. SNF2H was used as loading control. **b** Immunoblot showing XPA expression levels in XPA-proficient and XPA-knockout (XPA KO) U2OS cells expressing GFP-tagged XPF. Tubulin was used as loading control. **c** Clonogenic UV survival assays of wild-type U2OS cells without any transgene (U2OS) and XPA-proficient and XPA KO U2OS cells expressing ERCC1–GFP. Results are plotted as average of three independent experiments, each performed in triplicate. **d** Clonogenic UV survival assays of XPA-proficient and XPA KO U2OS cells expressing XPF–GFP. Results are plotted as average of three independent experiments, each performed in triplicate. In **c** and **d** error bars represent the SEM

To investigate how the activity of ERCC1–XPF in response to ICLs is regulated in cells, we studied how the complex is recruited to sites of locally induced psoralen-DNA crosslinks independently of NER. ERCC1–XPF binding to damaged DNA during NER critically depends on its interaction with XPA [15–19]. Therefore, we generated XPA-knockout (KO) cell lines by transfecting a plasmid encoding Cas9 and an sgRNA targeting exon 1 of XPA in previously generated U2OS cells expressing ERCC1–GFP [38] and U2OS XPF KO cells expressing XPF–GFP [37]. Sequencing and Tracking Indels by DEcomposition analysis [40] revealed multiple indel mutations in the *XPA* gene of a selected ERCC1–GFP-expressing and a selected XPF–GFP-expressing XPA KO clone (data not shown). The absence of XPA in these clones was confirmed by immunoblot (Fig. 1a, b), while strong UV hypersensitivity (Fig. 1c, d) showed that these cells indeed lack functional NER.

**Fig. 2:**
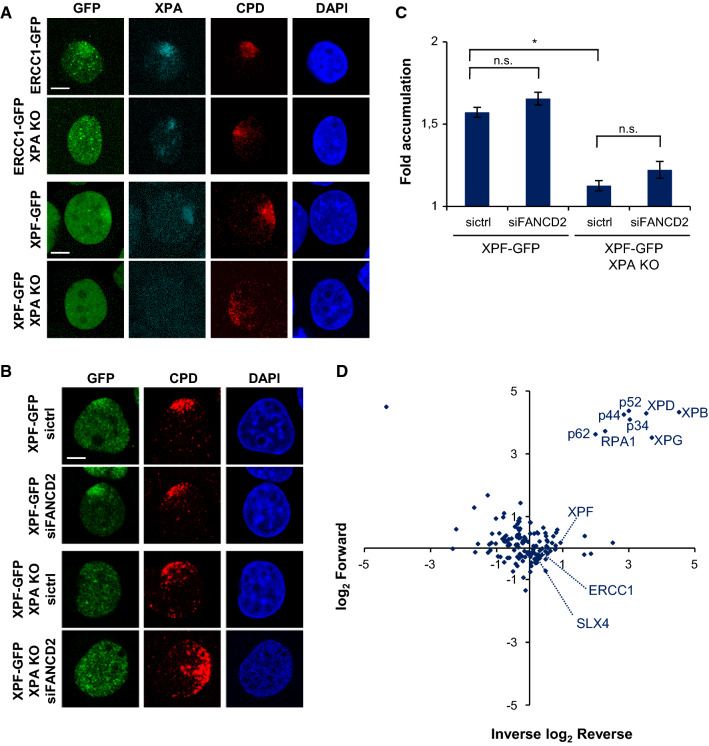
XPF recruitment to LUD is XPA dependent. **a** Immunofluorescence images showing LUD recruitment of ERCC1–GFP (top panel) or XPF–GFP (lower panel) and endogenous XPA in XPA-proficient and XPA KO U2OS cells, 1 h after 60 J/m^2^ UVC irradiation through an 8-µm microporous filter. Cells were immunostained against XPA and CPD, as damage marker. The GFP signal was not amplified using antibody staining. Scale bar: 5 µm. **b** Immunofluorescence images showing XPF–GFP LUD recruitment 1 h after 60 J/m^2^ UVC through an 8-µm microporous filter in XPA-proficient and XPA KO U2OS cells treated with nontargeting siRNA (sictrl) or siRNA targeting FANCD2 (siFANCD2). Cells were immunostained against GFP and CPD, which was used as damage marker. Scale bar: 5 µm. **c** Quantification of XPF–GFP LUD recruitment in XPA-proficient and XPA KO cells, as determined by immunofluorescence experiments shown in **b**. The fold accumulation at sites of local damage was calculated over the nuclear background and plotted as average of at least 100 cells per condition from two independent experiments. Statistical significant difference (*p* < 0.05) is indicated by asterisk, *n.s.* non-significant. **d** Scatter plot of the log_2_ SILAC ratio of the forward and reverse SILAC ERCC1–GFP immunoprecipitations experiments, comparing ERCC1–GFP interactors with and without UVC treatment (20 J/m^2^, 1 h). UV-specific interactors are depicted in the upper right quadrant. In **b** error bars represent the SEM

To show the validity of these cells in discerning between ERCC1–XPF activities in NER and in ICLR, we tested ERCC1–GFP and XPF–GFP recruitment to local UV damage (LUD) in XPA-proficient and XPA KO cells by immunofluorescence, after UVC irradiation through an 8-µm microporous filter (Fig. 2a–c). While both ERCC1 and XPF were clearly recruited to LUD in XPA-proficient cells, we could not observe clear recruitment in XPA KO cells. Depletion of FANCD2 in XPA-proficient and XPA KO cells expressing XPF–GFP, by a 48-h siRNA treatment (Fig. S1A), did not reduce XPF–GFP LUD recruitment as compared to control siRNA-treated cells (Fig. 2b, c). Our data confirm that ERCC1–XPF engagement in NER is completely dependent on XPA [45] and not regulated by FANCD2, indicating that the XPA KO cells can be used to study ERCC1–XPF activity outside NER.

To determine whether any additional, as-of-yet unidentified factors regulate ERCC1–XPF recruitment to UV damage, we combined ERCC1–GFP immunoprecipitation with stable isotope labeling by amino acids in cell culture (SILAC)-based quantitative proteomics (including label swapping, i.e., “forward” and “reverse” replicate experiments). To this end, SILAC-labeled ERCC1–GFP-expressing U2OS cells were mock-treated or exposed to UVC (20 J/m^2^) and 1 h after treatment chromatin-enriched nuclear extracts were subjected to GFP-mediated immunoprecipitation and mass spectrometry analysis. Interestingly, and as expected, this showed that after UV irradiation, ERCC1–XPF interacts with multiple components of the TFIIH complex, RPA and XPG (Fig. 2d and Table 1). Also XPA was identified as an interactor of ERCC1, but only in one of the replicate experiments and, therefore, not depicted in Fig. 2d. However, no additional new factors were identified, suggesting that no major or essential unknown factors exist that regulate ERCC1–XPF involvement in NER. Moreover, SLX4 was identified as a constitutive interaction partner of the endonuclease complex, in line with previous observations [2, 25, 26]. However, the interaction ratio of this partner did not change upon UV irradiation, in line with its specific role in ICLR.

**Table 1:** UV-dependent ERCC1–GFP-interacting proteins identified by SILAC-MS

Protein names	Unique peptides	Ratio H/L forward	Ratio L/H reverse	Average
XPB/ERCC3	18	22.991	20.10	21.55
XPD/ERCC2	24	11.55	19.57	15.56
p52/GTF2H4	8	8.0175	20.70	14.36
XPG/ERCC5	14	12.985	11.45	12.22
p34/GTF2H3	11	8.1806	17.08	12.63
p44/GTF2H2	13	7.2274	19.04	13.14
XPA	2		9.14	9.14
RPA1	7	4.8773	13.23	9.05
p62/GTF2H1	11	3.9822	12.33	8.15
XPF/ERCC4	51	1.9116	1.13	1.52
ERCC1	21	1.3952	0.93	1.16
SLX4	27	1.1329	0.72	0.93

Unique peptides and normalized ratios of the forward and reverse experiments of the top interacting proteins with ERCC1 1 h after 20 J/m^2^ UV irradiation. Also, peptides and ratios for ERCC1, XPF and SLX4 are shown

*H* heavy, *L* light

### ICLR and NER core factors localize to psoralen-induced DNA damage

**Fig. 3:**
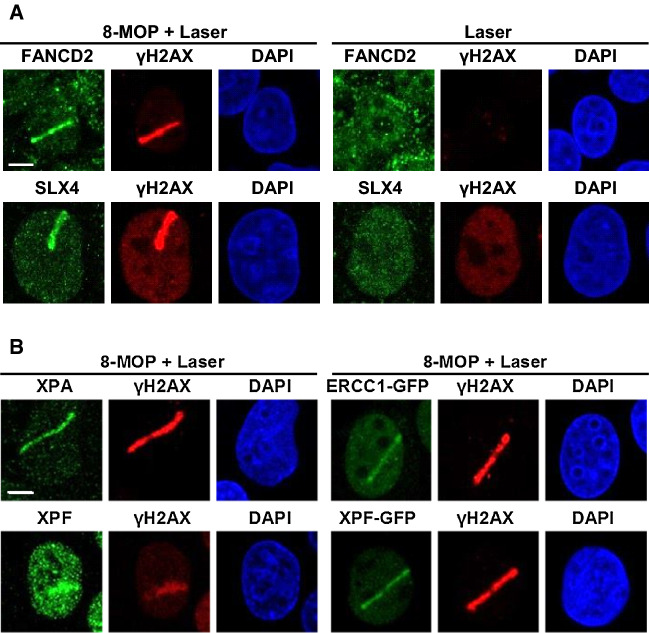
ICLR and NER core factors are recruited to psoralen-induced DNA damage. **a** Immunofluorescence images showing recruitment of endogenous FANCD2 and SLX4 to sites of local UVA laser microirradiation in untreated (laser) or psoralen-treated (8-MOP + laser, 50 µM 8-MOP, 2 h) U2OS cells. Cells were immunostained against FANCD2, SLX4 and γH2AX, which was used as damage marker. Scale bar: 5 µm. **b** Immunofluorescence images showing recruitment to sites of local UVA laser microirradiation in psoralen-treated U2OS cells (8-MOP + laser, 50 µM 8-MOP, 2 h) of endogenous XPA and XPF (left panels) and in psoralen-treated U2OS cells expressing ERCC–GFP or XPF–GFP (right panels). Cells were immunostained against XPA, XPF, GFP and γH2AX, which was used as damage marker. Scale bar: 5 µm

To analyze ERCC1–XPF recruitment to ICLs, we combined the use of the psoralen 8-methoxypsoralen (8-MOP) with UVA laser microirradiation along a user-defined track across the cell nucleus [44, 46], to activate the 8-MOP and induce local psoralen–DNA crosslinks. UVA activation of intercalated 8-MOP generates both monoadducts as well as ICLs in DNA [47–51]. The efficiency of this method to induce ICLs was shown by the clear recruitment of endogenous FANCD2 and SLX4 to local psoralen adducts, marked by γH2AX co-staining, as visualized by immunofluorescence in U2OS cells (Fig. 3a, 8-MOP + laser). Importantly, we did not observe recruitment of FANCD2 or SLX4 or induction of γH2AX in cells that were only UVA microirradiated but not treated with 8-MOP (laser), indicating that the applied UVA laser by itself did not generate DNA damage that leads to γH2AX signaling or is recognized by FANCD2. Furthermore, we observed clear recruitment of XPA to local psoralen adducts, as marked by γH2AX (Fig. 3b), indicative of targeting of the core NER machinery to psoralen adducts and suggesting the induction of NER substrate lesions. As monoadducts formed by psoralen are known NER substrates [52, 53], XPA is likely recruited to psoralen monoadducts as part of the NER machinery. Since NER is also implicated in replication-dependent and -independent ICLR [31, 32], XPA may be recruited to psoralen-ICLs as well. Moreover, also endogenous XPF as well as ERCC1–GFP and XPF–GFP were clearly recruited to psoralen adducts. Because of the induction of multiple types of DNA damage, this accumulation likely reflects the engagement of ERCC1–XPF in both active NER as well as ICLR.

### ERCC1–XPF recruitment to psoralen adducts is XPA and FANCD2 dependent

**Fig. 4:**
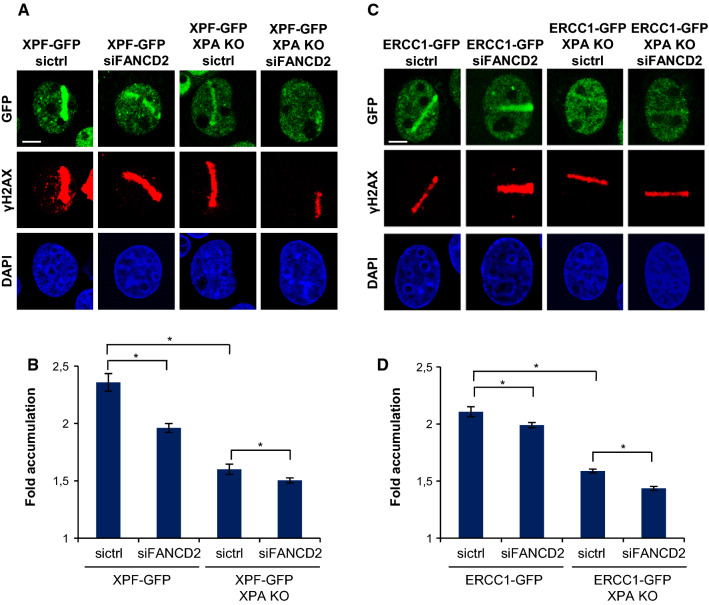
ERCC1–XPF recruitment to psoralen-induced DNA damage is XPA and FANCD2 dependent. **a** Immunofluorescence images showing XPF–GFP recruitment to sites of local UVA laser microirradiation in psoralen-treated (50 µM 8-MOP, 2 h) XPA-proficient and XPA KO U2OS cells treated with nontargeting siRNA (sictrl) or siRNA targeting FANCD2 (siFANCD2). Cells were immunostained against GFP and γH2AX, which was used as damage marker. Scale bar: 5 µm. **b** Quantification of XPF–GFP recruitment to sites of local UVA laser microirradiation in psoralen-treated XPA-proficient and XPA KO U2OS cells, as determined by immunofluorescence experiments shown in **a**. The fold accumulation at sites of local damage was calculated over the nuclear background and plotted as average of at least 80 cells per condition from three independent experiments. Statistically significant difference (*p* < 0.05) is indicated by asterisk. **c** Immunofluorescence images showing ERCC1–GFP recruitment to sites of local UVA laser microirradiation in psoralen-treated (50 µM 8-MOP, 2 h) XPA-proficient and XPA KO U2OS cells treated with nontargeting siRNA (sictrl) or siRNA targeting FANCD2 (siFANCD2). Cells were stained against GFP and γH2AX, which was used as damage marker. Scale bar: 5 µm. **d** Quantification of ERCC1–GFP recruitment to sites of local UVA laser microirradiation in psoralen-treated XPA-proficient and XPA KO U2OS cells, as determined by immunofluorescence experiments shown in **c**. The fold accumulation at sites of local damage was calculated over the nuclear background and plotted as average of at least 100 cells per condition from two independent experiments. Statistically significant difference (*p* < 0.05) is indicated by asterisk. In **b** and **d,** error bars represent the SEM

To test whether ERCC1–XPF recruitment to ICLs is dependent on FANCD2, we studied the recruitment of the complex to local psoralen adducts in ERCC1–GFP- and XPF–GFP-expressing XPA-proficient and -KO cells, depleted of FANCD2 by siRNA (Fig. 4). In XPA-proficient cells, XPF–GFP recruitment to psoralen adducts was reduced after siFANCD2 treatment compared to control siRNA-treated cells (Fig. 4a, b). Also, ERCC1–GFP recruitment was slightly reduced in siFANCD2-treated cells compared to control siRNA-treated cells (Fig. 4c, d). This was less pronounced than for XPF, possibly because of competition with endogenous ERCC1 that is still present in these cells (and which we did not visualize). These observations suggest that ERCC1–XPF recruitment to psoralen adducts is partially dependent on FANCD2. Furthermore, the absence of XPA also induced a strong reduction in ERCC1–GFP and XPF–GFP localization to psoralen adducts that was further reduced by additional FANCD2 depletion (Fig. 4). Our data, therefore, suggest that, in response to psoralen adducts, ERCC1–XPF is recruited both by XPA to participate in NER and by FANCD2 to function in ICLR. These observations in human cells are in accordance with chromatin immunoprecipitation (ChIP) studies in *Xenopus* egg extracts suggesting that FANCD2 preceeds and is needed for ERCC1–XPF recruitment to an ICL [2].

The stronger reduction in ERCC1–XPF recruitment due to XPA KO as compared to siFANCD2 could be because of incomplete FANCD2 depletion by siRNA. However, considering that we obtained efficient FANCD2 knockdown (Fig. S1a) this is likely better explained by the observation that psoralen-ICLs are preferentially repaired via unhooking by the DNA glycosylase NEIL3, which is independent of FANCD2–FANCI [30]. Moreover, XPA, but not FANCD2, is implicated in replication-independent ICLR as well [31, 33, 35]. Knockout of XPA will, therefore, also impair ERCC1–XPF engagement in this pathway. Importantly, this implies that even though in our experiments we did not distinguish between different cell cycle phases of the cells under study (which were rapidly dividing U2OS cells), the FANCD2-dependent recruitment of ERCC1–XPF reflects its engagement in replication-dependent ICLR. Finally, we observed residual ERCC1–XPF recruitment even in the absence of both XPA and FANCD2. It is thus possible that the ERCC1–XPF complex can still be targeted to psoralen adducts, albeit less efficient, even in the absence of both of these factors. Alternatively, it could be that this residual targeting reflects a function of the complex in yet another DNA repair pathway, possibly the repair of DSBs [5].

### R689S and S786F mutant XPF recruitment to psoralen adducts is XPA dependent

Several of the XPF mutations reported in patients have been described to disrupt ICLR. Among these are C236R, located in the SF2 helicase-like domain and associated with XPCS complex with FA features [7]; R689S, located in the nuclease domain and associated with FA [8]; S786F, also located in the nuclease domain and identified in a breast cancer [54]. Cells carrying the R689S or S786F mutant XPF alleles are hypersensitive to MMC but not to UV irradiation, suggesting that the R689S and S786F mutations only impair XPF function in ICLR [8, 37, 54]. Conversely, C236R renders cells hypersensitive to both MMC and UV irradiation, indicating that this mutation impairs XPF function in both ICLR and NER [7, 37]. Moreover, previous incision assays and ChIP experiments in *Xenopus* egg extracts showed that all three mutations impair the unhooking of a sequence-specific cisplatin–DNA ICL by XPF but not its recruitment to this lesion [55]. Therefore, we determined whether in human cells these mutations affect ERCC1–XPF recruitment to psoralen adducts.

**Fig. 5:**
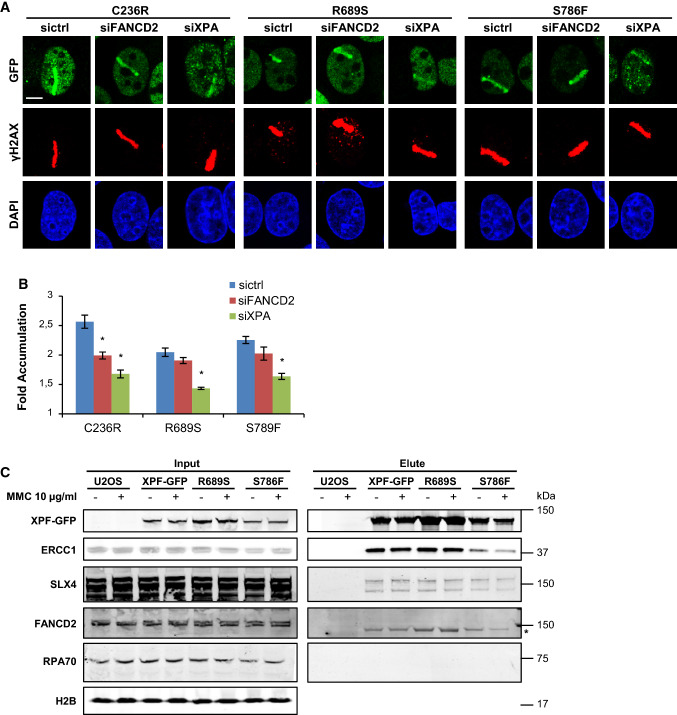
R689S and S786F mutant XPF is recruited to psoralen adducts in an XPA-dependent and FANCD2-independent manner. **a** Immunofluorescence images showing XPF recruitment to sites of local UVA laser microirradiation in psoralen-treated (50 µM 8-MOP, 2 h) U2OS XPF KO cells expressing GFP-tagged XPF carrying the amino acid mutations C236R, R689S or S786F, treated with nontargeting siRNA (sictrl) or siRNA targeting XPA (siXPA) or FANCD2 (siFANCD2). Cells were immunostained against GFP and γH2AX, which was used as damage marker. Scale bar: 5 µm. **b** Quantification of mutant XPF–GFP recruitment to sites of local UVA laser microirradiation in psoralen-treated U2OS XPF KO cells expressing GFP-tagged XPF carrying the amino acid mutations C236R, R689S or S786F, as determined by immunofluorescence experiments shown in **a**. The fold accumulation at sites of local damage was calculated over the nuclear background and plotted as average of at least 60 cells per condition from three independent experiments. Statistical significant difference (*p* < 0.05) compared to sicntrl of each cell line is indicated by asterisk. **c** Immunoblot analysis of cell lysate (input) and GFP immunoprecipitation samples (elute) from wild-type U2OS cells (U2OS) and XPF KO U2OS cells expressing GFP-tagged wild-type XPF (XPF–GFP) or XPF carrying the amino acid mutations R689S or S786F. Samples were analyzed with antibodies against GFP, ERCC1, SLX4, FANCD2, RPA70 and H2B (as loading control). Cells were mock treated (−) or incubated with 10 µg/ml MMC for 1 h before lysis. In the FANCD2 immunoblot, the asterisk indicates aspecific staining. In **b** error bars represent the SEM

We previously generated U2OS XPF KO cell lines that stably expressed GFP-tagged XPF mutants carrying the amino acid substitutions C236R (XPF–C236R), R689S (XPF–R689S) and S786F (XPF–S786F) [37]. To study their ability to be recruited to psoralen adducts repaired by NER or repaired by ICLR, we treated these cells with control, XPA and FANCD2 siRNA (Fig. S1a, B) and applied local UVA laser microirradiation after 8-MOP treatment. XPF–C236R was clearly recruited to sites of damage (Fig. 5a, b), which was reduced after FANCD2 and, even more, after XPA depletion. These results indicate that this XPF mutant is recruited to psoralen adducts as part of both the NER and the replication-dependent ICLR machinery, similarly as wild-type XPF (Fig. 4a, b). Conversely, XPF–S786F and, even more so, XPF–R689S were recruited less efficiently to psoralen adducts. Strikingly, their recruitment was only strongly reduced after XPA depletion but not significantly after FANCD2 depletion. This suggests that ERCC1–XPF carrying R689S or S786F can still be normally recruited as part of the NER machinery, in line with previous immunofluorescence and fluorescence recovery after photobleaching experiments that showed efficient binding of these mutants to UV-induced DNA damage [37]. However, the absence of a significant dependency on FANCD2 suggests that these mutants cannot efficiently be recruited to or stably associate with psoralen–DNA ICLs, which is in line with the MMC hypersensitivity of cells expressing these mutants [8, 37, 54]. Interestingly, the less efficient recruitment to psoralen adducts of XPF–R689S compared to XPF–S786F may indicate that this mutation has a more deleterious effect on XPF function in ICLR. This is in line with the stronger MMC hypersensitivity observed in cells expressing XPF with this mutation [37] and the stronger nuclease and ICL-unhooking defect found with purified *Xenopus* XPF carrying this mutation [55].

In an effort to understand why XPF–R689S and XPF–S786F are inefficiently recruited, we tested whether these XPF mutants showed reduced interaction with known or hypothesized XPF-interacting proteins. To this end, we performed GFP immunoprecipitation experiments on benzonase-treated lysate (i.e., to avoid isolation of DNA-mediated interactions/associations) from cells expressing GFP-tagged wild-type, R689S or S786F mutant XPF that was either mock treated or exposed to MMC. Importantly, we found that the mutant XPF proteins interacted normally with ERCC1 (Fig. 5c), which is in line with previous observations that these mutants stabilize, and thus bind ERCC1 [8, 37, 55]. Moreover, treatment with the crosslinking agent MMC did not alter the ratio of the interaction between XPF and ERCC1. It is currently unclear through which protein interactions ERCC1–XPF is recruited to ICLs, except that this depends on binding to its partner SLX4 [2, 25, 26], which was suggested to interact with mono-ubiquitylated FANCD2 [56]. Therefore, we tested whether these interactions were affected due to the XPF mutations. Strikingly, we did not observe any change in the interaction of XPF with SLX4, neither upon MMC exposure nor in the presence of the two XPF mutations. Moreover, we were unable to co-immunoprecipitate FANCD2 with XPF, suggesting that these proteins do not interact directly. Because in vitro ERCC1–XPF activity in ICLR is stimulated by RPA [27, 57], we also tested whether RPA directly interacts with wild-type or R689S or S786F mutant XPF. However, GFP-tagged XPF was unable to co-immunoprecipitate RPA70, the largest subunit of the heterotrimeric RPA complex, suggesting that stimulation of XPF activity by RPA is not via a direct physical interaction between the proteins. In summary, it remains unclear how exactly ERCC1–XPF, in complex with SLX4, is recruited to ICLs and how specific mutations in XPF could affect this. We observed that, even in the presence of both FANCD2 and XPA, recruitment of XPF–S786F and XPF–R689S is impaired. It was previously proposed that R689S and S786F affect the positioning of the XPF nuclease domain around the ICL rather than the actual catalytic activity itself [55, 58]. It is, therefore, possible that this incorrect positioning impairs a stable association of ERCC1–XPF with DNA at the lesion site, leading to inefficient visual recruitment at ICL sites as observed in our immunofluorescence experiments.

## Discussion

In this study, we show that in human cells, ERCC1–XPF is involved in the repair of the multiple types of lesions induced by psoralen–DNA crosslinking, as part of the NER machinery via XPA, and as part of replication-dependent ICLR via FANCD2. Our results highlight and help to better understand the multifaceted activity and regulation of ERCC1–XPF in DNA repair, which is essential for a better comprehension of how the multiple DNA repair pathways in which ERCC1–XPF is involved tightly collaborate to protect against cancer and promote health. Moreover, our data encourage to seriously consider the activation of multiple DNA repair pathways when evaluating the (side) effects of using DNA crosslinking agents as therapeutic drugs.

It would be interesting to further investigate the kinetics of ERCC1–XPF recruitment to ICLs, to determine how this changes over time in accord with unhooking and repair of the lesions. Moreover, considering the different mechanisms involved in replication-dependent and replication-independent ICLR and the different activities that NER might have in both pathways, it would be useful to study the roles of ERCC1–XPF in these two different mechanisms by investigating how the complex is recruited to ICLs specifically in S-phase and non-S-phase cells. Additionally, the interaction and interdependency of ERCC1–XPF with other ICLR, NER and HR factors should be determined in a cell cycle phase-specific manner, to precisely elucidate the multistep processes that lead to ICL removal. Possibly, ICLR factors that we did not investigate or unknown factors influence the recruitment of ERCC1–XPF and could help explain why the recruitment of XPF–R689S and XPF–S786F to ICLs is inefficient.

### Electronic supplementary material

Below is the link to the electronic supplementary material.

Supplementary material 1 (PDF 99 kb)
