# Supplementary material for: ERCC1–XPF targeting to psoralen–DNA crosslinks depends on XPA and FANCD2
Source: Cell Mol Life Sci. 2019 Aug 7;77(10):2005–16. doi: 10.1007/s00018-019-03264-5 (PMC7228994; doi:10.1007/s00018-019-03264-5)
Supplement: Supplementary file 1 — Supplementary material 1 (PDF 99 kb) [file 18_2019_3264_MOESM1_ESM.pdf]

Fig. S1

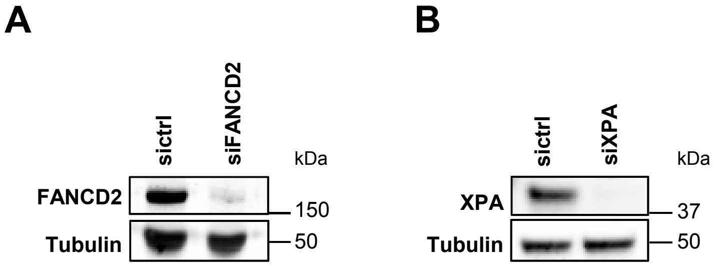

Fig. S1 ERCC1, XPF and XPA recruitment in ERCC1 and XPF wt or XPA KO cells. **a** Immunoblot showing FANCD2 expression in XPF KO U2OS cells expressing XPF-GFP treated with nontargeting control (sictrl) or FANCD2 siRNA (siFANCD2). Tubulin was used as loading control. **b** Immunoblot showing XPA expression in XPF KO U2OS cells expressing XPF-GFP treated with nontargeting control (sictrl) or XPA siRNA (siXPA). Tubulin was used as loading control.
